# Agreement between mothers’, fathers’, and children’s’ ratings on health-related quality of life in children born with esophageal atresia – a German cross-sectional study

**DOI:** 10.1186/s12887-019-1701-6

**Published:** 2019-09-11

**Authors:** Stefanie Witt, Janika Bloemeke, Monika Bullinger, Jens Dingemann, Michaela Dellenmark-Blom, Julia Quitmann

**Affiliations:** 1 Department of Medical Psychology, University Medical Center Hamburg-Eppendorf, Center for Psychosocial Medicine, Martinistraße 52, W 26, 20246 Hamburg, Germany; 2 Hannover Medical School, Center of Pediatric Surgery, Carl-Neuberg-Str. 1, 30625 Hannover, Germany; 3 Department of Pediatric Surgery, The Queen Silvia Children’s Hospital, 416 85 Gothenburg, Sweden

**Keywords:** Interrater-agreement, Health-related quality of life, Children, Parents, Self- and proxy-report, Rare disease, Esophageal atresia

## Abstract

**Background:**

Esophageal atresia (EA) is a rare congenital malformation, which is characterized by the discontinuity of the esophagus. We investigated the agreement between mothers’, fathers’, and children’s’ ratings on health-related quality of life (HRQOL) in children born with EA. We aimed to broaden the understanding of subjective experiences of HRQOL from different perspectives. We hypothesized that the agreement between mother and father ratings would be high, whereas the agreement between child and mother ratings as well as child and father ratings would show more substantial differences.

**Methods:**

We obtained data from 40 families (23 mother-father dyads of children aged 2–7 years and 17 mother-father-child triads of children and adolescents aged 8–18 years) with children born with EA, who were treated in two German hospitals. HRQOL was measured using the generic PedsQL™ questionnaires and the condition-specific EA-QOL© questionnaires. We calculated intraclass coefficients and performed one-way repeated measures ANOVAs to analyze differences for each domain as well as for the total scores.

**Results:**

Intraclass correlation coefficients (ICCs) indicated a strong agreement (≥.80) between mother and father reports of children’s HRQOL for both generic and condition-specific measurements. The ICCs for the generic HRQOL for mother/father-child-dyads revealed only fair to good agreement, whereas ICCs for condition-specific HRQOL showed high agreement for mother-child and father-child-agreement. Analyses of Covariance revealed differences in mother/father-child agreement in the generic domain School, both parents reporting lower HRQOL scores than the children themselves. Fathers reported significantly higher scores in the condition-specific domain Social than their children.

**Conclusions:**

Results showed that mothers’ and fathers’ reports corresponded to each other. Nonetheless, these reports might not be interchangeably used because mother-child and father-child agreement showed differences. Children might know the best on how they feel, and parent proxy-report is recommended when reasons such as young age, illness, or cognitive impairments do not allow to ask the child. But parent-report – no matter if reported by mother or father – should only be an additional source to broaden the view on the child’s health status and well-being. The current study contributes to a better understanding of the complex family relationships involved when parenting a child born with EA.

## Background

Esophageal atresia (EA) is a rare congenital malformation, which is characterized by the discontinuity of the esophagus. The prevalence varies between 1 and 2 in 5000 live births in Europe [1–3]. EA can be divided into different subtypes, with the majority of cases showing a connection between the esophagus and the windpipe. Most commonly, the affected children undergo surgical correction of the esophagus within a few days after birth. In more complicated cases, when the anatomic gap is too long, surgery may be delayed several months [1, 3, 4].

The prognosis on survival in infants born with EA has reached over 90% [1–4]. Nevertheless, long-term morbidity of EA patients remains both frequent and complicated. These complications are persisting into adulthood [5]. Studies demonstrate that morbidity among EA survivors results in swallowing difficulties [6], gastroesophageal reflux which can lead to symptoms such as vomiting, heartburn, cough and regurgitation [7–12] chronic and/or barky cough, wheezing and recurrent respiratory infections [13], as well as asthma, are frequently seen in patients [6, 13–15]. Additional morbidity can result from co-existing anomalies, which are present in approximately one-half of EA patients [16].

Accordingly, the disease and its treatment can have significant impacts on the individual and his/her family, and increased attention has been paid to the patient-reported outcomes (PRO's), especially the patients’ health-related quality of life (HRQOL).

HRQOL is an individual’s subjective perception of the impact of health status, including disease and treatment, on, e.g., physical, social, emotional functioning [17]. Dellenmark-Blom, Chaplin [18] have recently published a comprehensive review of the HRQOL of patients with EA. Accordingly, EA may be associated with reduced HRQOL, but several studies described inconsistent results about HRQOL of children born with EA.

Due to a lack of a cross-cultural available and valid instrument to measure HRQOL from the patients and parents perspective, a German-Swedish cooperation study was performed to develop and validate such a condition-specific instrument [19–22].

PRO’s, esp. HRQOL questionnaires are increasingly used in clinical practice and research for evaluation of the children’s health status [23]. In addition to medical information, knowledge of the HRQOL of a child born with EA is essential for treatment decisions, the assessment of the course of therapy, and the assessment of the therapeutic success [24, 25].

Since it is relevant to identify a reliable and valid source of information [26] the ongoing discussion on whom to ask for the assessment on children’s HRQOL is important from both the scientific and clinical perspective. Children are increasingly asked to provide a self-assessment of their HRQOL [27]. If the child is too young, ill, or due to cognitive impairments, the parent proxy-report is required, and usually the only source of information [24]. Children aged 8 or more can complete self-reports of HRQOL [28, 29] and it is important to give them the opportunity to report their own subjective experiences of health and well-being [30, 31], even if they need assistance in reading. Parental proxy-reports remain the most frequent source of additional information on children’s wellbeing and functioning [27].

In other pediatric health conditions, different studies have investigated the maternal/paternal report of children’s emotional and behavioral functioning, showing a stronger correspondence in parents than in other cross-informant pairs (such as parent-child, parent-teacher) [32–35].

Nevertheless, discrepancies are found between mothers' and fathers' ratings with again inconsistent findings: while some studies show that mothers focus more on symptoms/problems than fathers, other studies reveal no differences in problem severity between mothers’ and fathers’ ratings [35–37].

In usual pediatric settings, health care professionals and researchers often meet only one parent, it is less common to meet both parents. In the majority, mothers are usually the prime informant [38]. Still, the need to examine mother-father (dis-) agreement of children’s HRQOL aligns with the broader need to more regularly include fathers in child development and psychopathology research [35]. However, it is well known that children’s self-report and parent proxy-report of HRQOL are not interchangeable [39–41] and imperfect agreement between children and parents has been consistently demonstrated in the HRQOL measurement of children with chronic health conditions and healthy children [31, 42–44].

There are very few studies available that include the perspectives of children, mothers, and fathers to assess the children’s HRQOL and the understanding of mother and father agreement or discrepancies of their children’s HRQOL is needed for a comprehensive and entire view of the child’s HRQOL. Therefore, we aimed to assess the inter-rater-agreement in a clinical sample of children and adolescents born with EA for mother, father, and child ratings of generic and condition-specific HRQOL. On the one hand, we hypothesized that the agreement between mother and father ratings would be high, whereas the agreement between child and mother ratings as well as child and father ratings would show more significant differences. On the other hand, we expected differences regarding the level of agreement depending on the investigated HRQOL domains.

## Methods

The study was a part of a cross-cultural Swedish-German project aiming to develop a condition-specific questionnaire to measure HRQOL in children and adolescents born with EA [19–21]. The Ethical Review Board of Hannover, Germany (2936–2015) approved the study. Within this specific part of the study, we focus on the parental (dis-) agreement of young (2–7 years) children born with EA and the (dis-) agreement of mothers, fathers, and children (8–18 years). All children and adolescents who were treated for EA prior to 2016 either at the Medical School Hannover (MHH) or the Child Hospital ‘Auf der Bult’ as well as their parents were invited to participate. Inclusion criteria included children being between 2 and 17 years old at assessment and being diagnosed with EA. Exclusion criteria were insufficient German language proficiency, missing contact data, and missing clinical data.

A total of 102 eligible families were contacted between September and November 2016. A local researcher contacted the families and gave them oral and written study information. After the family gave written study approval, medical records were reviewed for clinical data, and questionnaires were sent home to the family with pre-stamped envelopes for their replies. Parents provided written consent for children and adolescents under the age of 16 years. They received a maximum of three reminders to return the questionnaires to increase response rate.

### Data collection

We measured generic child self-reported and parent proxy-reported HRQOL using the PedsQL™ questionnaire [45]. The questionnaire includes 23 items, assessing Physical, Emotional, Social, and School Functioning, and can be calculated in the three summary scores (Total Scores, Physical Health Summary Score, and Psychosocial Health Summary Score) with higher scores indicating better HRQOL. In the current sample, Cronbach’s alpha values were ranging from .72 (Social Functioning, mother-report) to .89 (Physical Functioning, mother-report; Total Score, child-report).

To assess condition-specific HRQOL, we used the EA-QOL©-questionnaires developed by the authors [19–21]. The 17-item EA-QOL© questionnaire for parents of children 2–7 years of age includes three domains; Eating, Physical health & Treatment, and Social isolation & Stress. In the current sample, the domains for parents of younger children showed acceptable to good reliability with Cronbach’s alpha values ranging from .73 (Eating, father-report) to .88 (Physical, mother-report). The 24-item EA-QOL© questionnaires for children and parents of children 8–17 years of age includes four domains; Eating, Social relationships, Body perception, and Health & Well-being, additionally a Total Score can be calculated from all domains; higher scores indicated better HRQOL. Acceptable to good reliability was also found in the current sample for the 24-item EA-QOL© questionnaires, with Cronbach’s α values ranging from .74 (Eating, father-report) to .91 (Physical, mother-report).

Additionally, we requested the parents to answer questions on sociodemographic data. Clinical variables were provided from medical records.

### Data analysis

For all statistical analysis, we used the *IBM Statistical Package for Social Sciences Statistics* version 25 [46]. We calculated means and standard deviations (SD) for each HRQOL domain. Except for sociodemographic and clinical variables, we replaced missing values by the individual mean score for each variable if missing data were random and less than 30% of the values (EA-QOL©) respectively 50% of the values (PedsQL™). The proportion of missing values in the current sample was less than 20%.

To investigate the inter-parental (dis-) agreement, respectively mother-child and father-child agreement on domain levels and for the total scores on an individual level, we calculated intraclass correlation coefficients (ICCs). The level of agreement is preferable evaluated by the ICCs estimate’s 95% confidence interval (CI) [47]. ICC values < .40, were interpreted as poor agreement, between .40 and .59 as fair agreement, ICC values between .60 and .74 as good agreement and values ≥ .75 represented excellent agreement [48]. The significance level was set to be less than .05 (*p* ≤ .05).

We performed one-way repeated measures ANOVAs to analyze differences between mother and father mean scores, respectively mother and child mean scores, as well as father and child mean scores for each domain as well as for the total scores on a group level. We entered the perspectives (mother vs. father resp. mother vs. child and father vs. child) as the within-subjects factors and children’s age and children’s gender as covariates into the model.

## Results

The study sample for the present analyses included 80 parents of 40 children and adolescents born with EA aged between 2 to 18 years of age (23 mother-father dyads of children aged 2–7 years and 17 mother-father-child triads of children and adolescents aged 8–18 years).

The mean age for mothers was 40.7 years (± 7.78), for fathers 43.1 years (± 8.82). Higher education (12 years or even a university degree) was present in 69.2% of the mothers and 57.6% of the fathers. 65.8% of the mothers worked part-time, none full time. 90% of the fathers worked full time, 10% part-time. Out of the 40 children and adolescents, 22 (55%) were males aged between 2 and 18 years old (M_age_ = 8.03, ± 4.45). According to predefined criteria [19], 24 (60%) of the children showed a mild-moderate severity level of EA, 16 (40%) showed severe disease characteristics. Except for one family, both parents live together in a household with the child.

Means and standard deviations for all HRQOL domains are presented in Table 1. Almost all values score in the upper segment of the 0–100 transformed scales with means ranging from 64.08 (mother-reported generic HRQOL, Emotional, 2–7 years) to 91.41 (child-reported generic HRQOL, Physical, 8–18 years). Mother reported lower HRQOL than fathers in almost all domains; except the generic domains Physical and Social (2–7 years) as well as the condition-specific domain Eating (8–18 years). The lowest average HRQOL mean scores for mother-reported HRQOL appeared on the generic domain Emotional for 2–7 years-old children (M = 64.08), for father-reported HRQOL on the condition-specific domain Body and Health for 2–7 years-old children (M = 69.59) and for child-reported HRQOL the lowest mean scores appeared on the condition-specific domain Eating (M = 74.20).

**Table 1:** Mean scores, standard deviation and ANCOVA for repeated measures for mother, father and child-reported children’s HRQOL

HRQOL		Mother and father reports (*n* = 40)				Mother and child reports (*n* = 16)				Father and child reports (*n* = 16)			
		n	Mother	Father	*p*	n	Mother	Child	*p*	n	Father	Child	*p*
			M (SD)	M (SD)			M (SD)	M (SD)			M (SD)	M (SD)	
Generic													
2–7 yrs	Physical	23	80.93 (20.91)	78.51 (20.67)	.32		na	na			na	na	
	Social	23	81.52 (18.92)	80.80 (20.34)	.56		na	na			na	na	
	Emotional	23	64.08 (18.15)	71.03 (18.49)	.04*		na	na			na	na	
	School	22	71.52 (21.09)	75.91 (19.48)	.22		na	na			na	na	
	Psychosocial	23	71.63 (15.12)	75.69 (15.66)	.13		na	na			na	na	
	Total	23	74.72 (15.33)	76.59 (16.24)	.40		na	na			na	na	
8–18 yrs	Physical	16	84.38 (18.49)	88.37 (12.30)	.21	16	87.89 (11.85)	91.41 (11.44)	.29	16	90.54 (8.70)	91.41 (11.44)	.75
	Social	17	88.75 (14.43)	91.25 (10.72)	.30	16	88.75 (14.43)	88.44 (14.80)	.93	16	91.25 (10.72)	88.44 (14.80)	.46
	Emotional	16	81.84 (18.76)	83.97 (18.44)	.46	15	82.33 (18.21)	89.00 (17.55)	.10	15	86.00 (14.54)	89.00 (17.55)	.49
	School	17	71.47 (17.66)	68.24 (17.76)	.30	16	72.50 (17.70)	82.19 (12.78)	.02*	16	68.13 (18.34)	82.19 (12.78)	≤.01**
	Psychosocial	17	79.39 (16.49)	79.99 (13.16)	.75	15	81.78 (14.78)	87.00 (11.59)	.10	15	82.02 (10.91)	87.00 (11.59)	.07
	Total	17	81.11 (16.56)	82.91 (11.89)	.39	15	84.13 (13.20)	88.06 (10.46)	.18	15	85.27 (8.83)	88.06 (10.46)	.25
Condition-specific													
2–7 yrs	Eating	19	71.55 (13.27)	73.50 (13.75)	.45		na	na			na	na	
	Body	22	64.51 (26.73)	69.58 (25.71)	.06		na	na			na	na	
	Social	19	84.21 (21.89)	85.20 (22.56)	.77		na	na			na	na	
	Total	21	70.26 (20.58)	73.85 (19.53)	.12		na	na			na	na	
8–18 yrs	Eating	14	73.66 (18.21)	72.51 (18.86)	.69	14	73.66 (18.21)	75.70 (20.95)	.47	15	72.47 (18.18)	74.20 (21.01)	.54
	Social	14	80.36 (18.94)	86.22 (17.77)	.01**	14	80.36 (18.94)	78.32 (21.90)	.53	15	86.43 (17.14)	79.05 (21.30)	.03*
	Body	15	84.33 (22.43)	85.33 (19.13)	.60	15	84.33 (22.43)	91.00 (18.05)	.18	15	85.33 (19.13)	91.00 (18.05)	.16
	Health	15	87.50 (16.37)	88.33 (19.89)	.81	15	87.50 (16.37)	84.58 (23.25)	.50	15	88.33 (19.87)	84.58 (23.25)	.16
	Total	14	79.99 (16.51)	82.02 (16.61)	.14	14	79.99 (16.51)	80.98 (18.22)	.52	15	81.97 (16.01)	80.85 (17.56)	.48

Covariates included in the model: children’s age and children’s gender

*M* Mean, *SD* Standard deviation, *p P*-value, *na* Not applicable

* *p* ≤ .05; ** *p* ≤. 01

On a group level, analyses of covariance for repeated measures (ANCOVA) showed significant differences between mother- and father-reported children’s HRQOL for two HRQOL domains; generic domain Emotional for 2–7-years-old (*F* = 5.01, *p* = .04) and condition-specific HRQOL domain Social for 8–18-years-old (*F* = 8.59, *p* = .01). All other domains and total scores showed no significant differences between mother and father-reported HRQOL. Differences between maternal and children’s mean scores on a group level were found for the generic domain School (*F* = 7.58, *p* = .02). For paternal and children’s mean scores, our analysis showed significant differences for the generic domain School (*F* = 15.26, *p* ≤ .01) and the condition-specific domain Social (*F* = 5.57, *p* = .03) (Table 1).

On an individual level, ICCs indicated a strong agreement (≥.80) between mother and father reports of children’s HRQOL for both generic and condition-specific measurements. The lowest ICCs we found for the generic HRQOL domain Emotional (2–7-years-old) and the generic HRQOL domain Physical (8–18-years-old) with .80. The highest ICCs we found for the condition-specific domain Body (8–18-years-old) and the condition-specific Total score (8–18-years-old) with .97 (Table 2).

**Table 2:** Inter-rater reliability: intraclass correlation coefficients for mother-father agreement, mother-child agreement, and father-child agreement

HRQOL			Mother and father reports		Mother and child reports		Father and child reports	
			ICCs^a^	[CI]	ICCs^a^	[CI]	ICCs^a^	[CI]
Generic	2–7 yrs	Physical	.94**	[.85–.97]	na	na	na	na
		Social	.90**	[.77–.96]	na	na	na	na
		Emotional	.80**	[.50–.92]	na	na	na	na
		School	.91**	[.78–.96]	na	na	na	na
		Psychosocial	.87**	[.69–.95]	na	na	na	na
		Total	.93**	[.83–.97]	na	na	na	na
	8–18 yrs	Physical	.80**	[.47–.93]	.57	[−.18–.85]	.64*	[−.08–.86]
		Social	.84**	[.57–.95]	.70*	[.10–.90]	.49	[−.47–.82]
		Emotional	.89**	[.71–.96]	.78**	[.38–.93]	.66*	[−.03–.89]
		School	.86**	[.62–.95]	.67**	[.06–.88]	.60**	[−.17–.86]
		Psychosocial	.93**	[.82–.98]	.74**	[.34–.91]	.74**	[.25–.91]
		Total	.91**	[.76–.97]	.73**	[.24–.91]	.71*	[.18–.90]
Condition-specific	2–7 yrs	Eating	.81**	[.50–.93]	na	na	na	na
		Body	.94**	[.84–.98]	na	na	na	na
		Social	.88**	[.70–.96]	na	na	na	na
		Total	.93**	[.82–.97]	na	na	na	na
	8–18 yrs	Eating	.92**	[.75–.97]	.93**	[.79–.98]	.93**	[.78–.98]
		Social	.94**	[.67–.98]	.91**	[.73–.97]	.86**	[.74–.96]
		Body	.97**	[.92–.99]	.75**	[.25–.91]	.81**	[.44–.93]
		Health	.86**	[.57–.95]	.81**	[.43–.94]	.94**	[.83–.98]
		Total	.97**	[.93–.99]	.97**	[.91–.99]	.97**	[.91–.99]

*ICC* Intraclass correlation coefficient (two-way mixed model. Absolute agreement, *CI* 95% confidence interval, *na* Not applicable

* *p* ≤ .05; ** *p* ≤. 01

^a^ Intraclass correlation coefficients reference values: ICC < .40 = poor agreement, ICC between .41 and .60 = moderate agreement, ICC between .61 and .80 = good agreement, ICC > .81 = excellent agreement [49]

The agreement on individual level for mother-child-dyads showed excellent agreements for the condition-specific HRQOL measurement with the lowest ICCs for the domain Body & Health (.75) and the highest agreement for the Total Score (.97). The ICCs for the generic HRQOL for mother-child-dyads revealed only fair to good agreement. Only for the generic domain Emotional, the ICCs showed excellent agreement (.78). Analyzing father-child-dyads, we found high agreement for the condition-specific HRQOL measurement between .81 and .97. The father-child-agreement on generic HRQOL revealed fair to good results with the lowest ICCs for the generic domain Social (.49) (Table 2).

## Discussion

From our point of view, it is crucial to measure HRQOL from the perspectives of different family members. So far, only few research studies have included multiple family members, and to our knowledge, this is the first study aiming to investigate the agreement between mothers, fathers, and child-reported HRQOL in a sample of children born with EA. We assessed parental perceptions of HRQOL of their children born with EA as well as children’s perceptions. We compared mothers’ and fathers’ reports on children’s HRQOL as well as mothers’/fathers’ and children’s report to examine the level of agreement respectively differences using generic as well as condition-specific measurements.

Measuring congruence beyond dyadic relationships addresses this family science research gap and contributes to this body of literature because study findings suggest multiple family members do not always perceive HRQOL alike [50, 51].

It is challenging to define, conceptualize, interpret, measure, and improve HRQOL because HRQOL is subjective, and patients and parents identify fundamentals they feel are important to their HRQOL [52–55]. Studying the different perspectives from multiple family members involved in the children’s health care process is important because of the above-mentioned inconsistencies in the literature, which reveal that the children’s perception of HRQOL might vary from their family members’ perceptions. This variation has clinical (e.g., regarding the focus of treatment for children and each parent) as well as research implications (e.g., regarding possible differences on the definition of HRQOL the families, since we do not know if family members disagree on the definition of HRQOL or only on how to quantify it). Therefore, congruence regarding HRQOL is important to the long-term well-being of families with children suffering from rare chronic diseases, but parents and health providers should be aware of differences in the perception of HRQOL from different family members.

Results of our study showed strong inter-parental agreement on individual level across all dimensions for both generic and condition-specific HRQOL. On group level, we found no significant differences between maternal and paternal report. Only for the generic domain Emotional (2–7 years) and the condition-specific domain Social, we found a significant difference between mother and father report, showing that fathers reported higher HRQOL scores than mothers.

These high mother-father agreements are in line with current research, that reported moderate to strong agreements [35, 37, 50, 57–59]. But while most studies investigated internalizing and externalizing behavior, only two studies are available, that focused on inter-parental agreement of parent-reported child HRQOL [56, 60]. These samples consisted of parents of children with mental disorders in a clinical setting or parents of healthy children. Only one study included parents of chronically ill children [60].

Nonetheless, our findings of high inter-parental agreement do not imply that maternal and paternal reports might be used interchangeably.

The ICCs for mother-child-reports as well as father-child-reports revealed higher agreement for the condition-specific measurements, with an excellent agreement for all domains except mother-child agreement on the condition-specific domain Body Perception. However, the agreement for the generic HRQOL showed only moderate to good agreement. Contrary to current literature, the parent-child agreement did not show higher ICCs for the domain Physical [24, 41]. The different results of parent-child agreement depending on the used measurement might be explained by the need to communicate about disease-specific demands [61] and a high degree of parental awareness of disease-specific aspects of children’s daily life.

Both mothers and fathers tended to underrate the children’s generic HRQOL regarding the domain School. This domain assesses aspects of absentee days in school and the ability to concentrate on school works. This significant underrating of children’s HRQOL in the domain School shows that parents of children born with EA might assess the disease-specific effects of EA on the child’s daily life significantly higher than the children. Silva, Bullinger [62] reported significantly higher ICCs for parent-child agreement using chronic-generic compared to generic measurements. Parents seem to be more easily able to empathize with their child about condition-specific aspects of daily life. This might be because parents and children regularly discuss the disease and the stresses and challenges in everyday life. Aspects of generic HRQOL seem not to be as relevant as disease-specific aspects in family life. The HRQOL measurement should be selected and used depending on the target. Condition-specific questionnaires have the advantage to be more sensitive. Therefore, they should be used to evaluate quality of life changes in a clinical trial, after surgical treatment or psychosocial interventions. Generic HRQOL measurements allow the use in healthy as well as clinical populations regardless of the health condition status and enable comparison between different health conditions as well as comparisons with healthy reference populations [23].

Also, our results showed that fathers tended to overrate children’s condition-specific HRQOL in the domain Social. The domain Social assess aspects of bullying and social exclusion due to EA. Adolescents behave differently towards their parents and interpret parental behavior differently, depending on whether they interact with their mother or their father [63]. We assume that the differences in the parental perception of the condition-specific domain Social might be due to differences in the mother-child, respectively father-child relationship [64]. Adolescents might talk about experiences of bullying and social exclusion more often or more openly with their mothers [65].

Mothers and fathers of children and adolescents take on distinctive tasks regarding the development of their child [64, 66]. This might explain the differences between the agreement of mother-child-reports and father-child-reports. Although mothers are typically the primary caregiver when the child has special health care needs, fathers of children with developmental delays or physical illness are more highly involved in the daily care than fathers of healthy children [67]. Fathers of children with chronic diseases showed an increase of involvement [68, 69] and therefore the high agreement between mothers and fathers of our sample might be explained by high involvement and interest of the participating fathers in health and development of the children.

In summary, while more work remains, our results present the first evidence of high inter-parental agreement on children’s HRQOL in patients born with EA over almost all domains of HRQOL for both generic and condition-specific HRQOL. Since in our study, we examined only a small sample of patients born with EA and their parents, the results may only be interpreted as first tendencies. Still, there is not enough evidence that allows replacing mother and father reports even though the inter-parental agreement was high, esp. when using condition-specific HRQOL measurements. A systematic inclusion and evaluation of the triadic family system is necessary to get a comprehensive and entire view of the children’s health.

## Limitations

Since almost all families that participated had two parents living in the same household, findings of this study might not be representative (e.g., the situation single parents might differ). The stressors in this kind of families might be quantitatively as well as qualitatively different, considering that less communication might take place between both parents. Reports from mothers and fathers could also largely differ depending on who is looking after the child for which period. This aspect should be considered in future studies. Due to the small sample size, we were not able to control for the age of the child, child’s gender or severity level of disease.

Another limitation applying to the findings, in general, is that no control was undertaken on social desirability effects. For instance, parents might have reported a higher HRQOL than the actual one. This suggestion derives from the fact that the parents were recruited from the medical clinic at which their children are treated. Parents would probably be refrained from expressing negative attitudes that might indirectly involve the doctor; it would be rather more acceptable for them to report as if things are generally going moderately well. Since the families filled out the questionnaires in a home environment, we cannot exclude that parents and children coordinated their answers even though they had explicitly been asked not to talk to each other while answering the questionnaires. The results of this study can be used as an indication for future studies investigating the triadic agreement between mothers, fathers, and children with rare malformations such as EA.

## Conclusions

The findings of the present study are important in the context of providing information on HRQOL of German children born with EA from mothers’, fathers’, and children’s’ perspectives.’ Results show that mothers’ and fathers’ reports correspond to each other.

Nonetheless, these reports might not be interchangeably used because of mother-child and father-child agreement reveal differences. Children might know the best on how they feel, and parent proxy-report is recommended when reasons such as young age, illness, or cognitive impairments do not allow to ask the child. But parent-reports – no matter whether mother or father – should only be an additional source to broaden the view on the child’s health status and well-being.

To conclude, the current study contributes to the different perspectives when parenting a child born with EA. The study’s unique contributions include an examination of the strong inter-parental agreement on HRQOL involving multiple members’ perspectives. This provides empirical evidence for how three family members with different perceptions can be combined to study the “whole” family and HRQOL.

## Data Availability

The datasets used and/or analyzed during the current study are available from the corresponding author on reasonable request.

## Abbreviations

ANOVA: Analysis of variance
CI: Confidence interval
EA: Esophageal atresia
EA-QOL©: Esophageal Atresia – Quality of Life Questionnaires
F: *F*-value
HRQOL: Health-related quality of life
ICC: Intraclass correlation coefficient
M: Mean
MHH: Medical School Hannover
p: *P*-value
PedsQL™: Pediatric Quality of Life Inventory TM
PROs: Patient-reported outcomes
SD: Standard deviations
UKE: University Medical Center Hamburg-Eppendorf
